# Product/Process Fingerprint in Micro Manufacturing

**DOI:** 10.3390/mi10050340

**Published:** 2019-05-22

**Authors:** Guido Tosello

**Affiliations:** Department of Mechanical Engineering, Technical University of Denmark, Produktionstorvet, Building 427A, 2800 Kgs. Lyngby, Denmark; guto@mek.dtu.dk; Tel.: +45-45-25-4893

The continuous trend towards miniaturization and multi-functionality embedded in products and processes calls for an ever-increasing research and innovation effort in the development of micro components and related micro manufacturing technologies. Highly miniaturized systems manufactured by a wide variety of materials find applications in key technological fields such as healthcare devices, micro implants, mobility and communications sensors, optical elements, and micro electromechanical systems.

High-precision and high-accuracy micro component manufacturing can be achieved through post-process (i.e., off-line) and in-process (i.e., on-line) metrology of both process input and output parameters, as well as the geometrical and functional features of the produced micro components. It is of critical importance to reduce metrology and optimization efforts, since process and product quality control can take a significant portion of total production time and cost in micro manufacturing.

To solve this fundamental challenge, research efforts are undertaken in both the industrial and scientific communities to define, investigate, implement and validate the so-called “Product/Process Manufacturing Fingerprint” concept.

The “Product Manufacturing Fingerprint” refers to that unique dimensional and/or functional outcome (e.g., surface topography, form error, etc.) on the produced component that, if kept under control and within specifications, ensures that the entire micro component complies with its specifications.

The “Process Manufacturing Fingerprint” is a specific process parameter or feature to be monitored and controlled in order to maintain the production of products complying with their specifications. Effective process monitoring will control the presence of a specific “Process Manufacturing Fingerprint” based on relevant process variables that are measured by sensors. This allows for real-time process control aiming at zero-defect micro manufacturing of the “Product Micro Fingerprint”, and as a consequence of the whole component. By integrating both Product and Process Manufacturing Fingerprint concepts, metrology and optimization efforts are highly reduced and the micro product quality increased, with an obvious improvement of the production yield.

Accordingly, this Special Issue seeks to present research papers focusing on innovative developments and applications in precision micro manufacturing process monitoring and control, as well as microproduct quality assurance and characterization. The focus will be on micro manufacturing process chains and their Product/Process Fingerprint, towards full process optimization and zero-defect micro manufacturing.

The Special Issue consists of 16 original research papers, which cover both fundamental process technology developments, as well as their application, combined with the definition and the validation of Product and Process Manufacturing Fingerprints.

The papers included in the Special Issue address research in two main areas of the Micro Manufacturing Fingerprint:

(1) Definition and application of Product Fingerprints for new product development and functional characterization.

- In the field of micro optics, Wang et al. [1] presented a study on the development of single composite diffractive optical element for color images generation, and Cao et al. [2] worked on the design and fabrication of an artificial compound eye for multi-spectral imaging.
- In the field of micro mechanical systems and sensors, Díaz Pérez et al. [3] presented the development of a one-dimensional control system for a linear motor of a two-dimensional nanopositioning stage, and Choi et al. [4] showed how the vibration acceleration can be used as Product Fingerprint on the development of a miniaturized mobile haptic actuator.

(2) Definition and application of the Process Fingerprints for process development, monitoring and control.

- In the field of injection molding of micro structured components, Giannekas et al. [5] and Loaldi et al. [6] presented studies concerning quality assurance and process control for the manufacturing of polymer microfluidic systems and Fresnel lenses, respectively. With regards to micro injection molding, Baruffi et al. [7] and Luca et al. [8] presented studies related to the correlation between Process-related Fingerprints and the achieved Product Fingerprints (flash marks and flow length, respectively).
- As far as machining processes are concerned, Process Fingerprints were established and validated for electrical discharge machining (EDM) by Świercz et al. [9], for micro EDM drilling by Bellotti et al. [10], for plasma electrolytic polishing by Danilov et al. [11], for jet electrochemical machining by Yahyavi Zanjani et al. [12], for nanosecond pulsed laser ablation by Cai et al. [13], and for micro grinding by Fook et al. [14].
- Process Fingerprints were developed for forming and additive processes by Cannella et al. [15] and Guo et al. [16], respectively. The former presented process development and monitoring of electro sinter forging for the manufacturing of miniaturized titanium discs and rings. The latter monitored and characterized electrohydrodynamic jet (e-jet) printing for the establishment of an optimized process window.

We wish to thank all authors who submitted their papers to the Special Issue “Product/Process Fingerprint in Micro Manufacturing”. We would also like to acknowledge all the reviewers whose careful and timely reviews ensured the quality of this Special Issue.

A special thanks is due to all the colleagues of the European project MICROMAN (“Process Fingerprint for Zero-defect Net-shape MICROMANufacturing”, http://www.microman.mek.dtu.dk/, 2015–2019). MICROMAN is a Marie Skłodowska-Curie Action Innovative Training Network supported by Horizon 2020, the European Union Framework Programme for Research and Innovation (Project ID: 674801). The support and funding from the European Commission is highly appreciated.
